# The A431E PSEN1 mutation arising as a founder effect in Jalisco State in Mexico: clinical features, imaging, and neuropathological findings

**DOI:** 10.1002/alz70857_098880

**Published:** 2025-12-24

**Authors:** John M Ringman

**Affiliations:** ^1^ Keck School of Medicine, University of Southern California, Los Angeles, CA, USA; Alzheimer's Disease Research Center, Keck School of Medicine, University of Southern California, Los Angeles, CA, USA; University of Southern California, Los Angeles, CA, USA

## Abstract

**Background:**

Autosomal dominant Alzheimer's disease (ADAD) is a rare subtype of AD in which the disease is caused by mutations in the PSEN1, APP, or PSEN2 genes which are essentially fully penetrant. Mutations in PSEN1 are most common and several populations have been described within which the same causative variant appears to have arisen from a common founder. The study of such populations allows for an increased sensitivity for identification of genetic and non‐genetic variables affecting phenotype and for studying the effects of interventions.

**Method:**

Review of the literature on the A431E PSEN1 mutation.

**Result:**

Over the last 20 years collaborators in the U.S and Mexico have seen over 100 superficially distinct families with the common A431E substitution in *PSEN1*, apparently arising as a founder effect concentrated in the Los Altos region of Jalisco State. With a mean age of onset of 41 years, approximately 40% of affected persons feature significant leg stiffness (spastic paraparesis) as an early symptom. Cognitive features can be that of a typical onset of episodic memory problems though disproportionate language problems or a dysexecutive syndrome can occur. Preliminary data show that this mutation is associated with severe cerebral amyloid angiopathy (CAA) and atypical “cotton wool” amyloid plaques on pathology. Flortaucipir PET scans shows atypical involvement of primary motor and parieto‐occipital cortex and diffusion MRI shows widespread abnormalities in white matter ultrastructure.

**Conclusion:**

As the number of persons at‐risk for inheriting it has been estimated at 1,260, the A431E mutation in *PSEN1* is an attractive target for both mutation‐specific genetic interventions and for anti‐AD therapy in general.

Supported by P30AG066530, U01AG051218, R01AG062007, R01AG069013

